# Histopathological lesions in reproductive organs, distal spinal cord and peripheral nerves of horses naturally infected with *Trypanosoma equiperdum*

**DOI:** 10.1186/s12917-019-1916-7

**Published:** 2019-05-28

**Authors:** Ahmed Yasine, Hagos Ashenafi, Peter Geldhof, Leen Van Brantegem, Griet Vercauteren, Merga Bekana, Alemu Tola, Ann Van Soom, Luc Duchateau, Bruno Goddeeris, Jan Govaere

**Affiliations:** 1 Wollo University, School of Veterinary Medicine, P.O Box 1145, Dessie, Ethiopia; 2 Addis Ababa University, College of Veterinary Medicine, P.O Box 34, Bishoftu, Ethiopia; 3 Ghent University, Faculty of Veterinary Medicine, 9820 Merelbeke, Belgium; 4 Vet-Path bvba, 9991 Adegem, Belgium; 5 Katholieke Universiteit Leuven, Faculty of Bioscience Engineering, 3001 Heverlee, Belgium

**Keywords:** Dourine, T. Equiperdum, Horses, PCR, Histopathology

## Abstract

**Background:**

Dourine, a venereal transmitted trypanosomosis caused by *Trypanosoma equiperdum*, has different clinical signs related to the reproductive and nervous system. Pathologic tissue changes associated with the disease are poorly described. The present study describes the histopathological lesions in naturally *T. equiperdum*-infected horses in the chronical stage of dourine.

**Results:**

Four chronically dourine diseased horses underwent a post-mortem examination. They were Woo test negative, but CATT*/T. evansi* positive, had a low packed cell volume (PCV) and exhibited obvious clinical signs of dourine. Post-mortem examination did not reveal gross lesions in the organs assumed to be responsible for the symptomatology. On histopathology, genital organs were affected, with mononuclear cell infiltration and erosions and degeneration of seminiferous tubules and perivascular lymphoplasmacytic cuffing in the uterus. In the nervous system, mononuclear cell infiltration was located in peripheral nerves, ganglia and in the spinal cord, leading to axonal degeneration. Real-time PCR using ITS primer revealed the presence of trypanosomes in these organs and conventional PCRs using maxicircle and RoTat1.2 primers further confirmed the involvement of *T. equiperdum* since the DNAs from the vagina, testicle, distal spinal cord, sciatic and obturator nerves found to be positive for maxicircle and negative for RoTat 1.2.

**Conclusions:**

The histopathological lesions in the spinal cord and peripheral nerves explain the incoordination of the hind legs in *T. equiperdum*-infected horses, whilst its presence in the genital tract exemplifies the venereal transmission.

## Background

Ethiopia has a population of more than 2.1 million horses [1]. Horses play a prominent role in agricultural and transport systems [2, 3]. Agricultural operations depend predominantly on manual labour and horses are the main means to transport both people and products [4]. Among the multiple health and welfare problems affecting equids, parasitic diseases are a major constraint to their productivity and often lead to high morbidity and mortality [5]. Dourine is an important parasitic disease affecting horses [6–9] and is caused by *Trypanosoma equiperdum*. In contrast with other trypanosomes, *T. equiperdum* is not transmitted by an invertebrate vector but through coitus [10]. The disease is characterized by acute oedematous swelling of the genitalia and cutaneous plaques followed by chronic fatigue, incoordination of hind legs and emaciation leading to death [7, 11].

Diagnosis of dourine by parasitological techniques is difficult due to the low parasitaemia in chronically infected animals. Therefore in endemic areas, demonstration of trypanosomal antibodies and the presence of symptoms is used to diagnose the disease [7–9, 12–14].Characterization of the origin and treatment options of dourine were reported [15–17], however, necropsy finding of diseased horses were not clearly nor thoroughly described. The pathological changes and detection of the infectious agent in different organs of *T. equiperdum* infected animals associated with the clinical signs have not yet been clarified. Therefore the objective of this study was to describe the histopathological lesions of naturally infected horses at the chronic stage of dourine.

## Results

### Symptoms

**Fig. 1:**
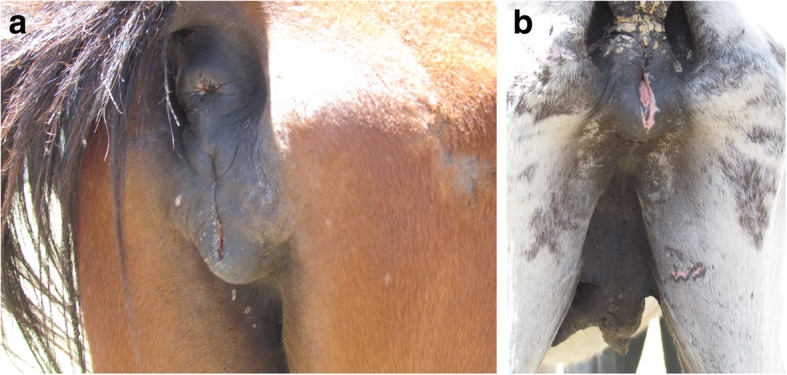
Symptoms in dourine diseased mares: Swollen vulva (**a**) Ulcerated lesion of labia and oedema of the mammary gland in non-lactating mare (**b**)

Horses positive for dourine exhibited various characteristic clinical signs. Animals were emaciated, had an inelastic skin and a dull hair coat. Horses did not suffer a loss of appetite but body weight loss aggravated leading to cachexia. Oedema of the external genitalia with vaginal discharge (Fig. 1a), ulceration (Fig. 1b) and depigmentation of perineal skin were prominent signs in mares. One of the mares showed oedema of the mammary glands (Fig. 1b) with watery secretion. Nervous signs such as a staggering movement and hindquarter ataxia were observed. Clear symptoms involving the genital system of the stallions were not noticed except depigmentation on the penile skin.

### Parasitology and serology

During the selection process, attempts to isolate the parasite in the buffy coat using the Woo test from clinically and serologically (CATT*/T. evansi*) positive horses were unsuccessful and no trypanosomes were detected in any of the samples. The clinical signs observed in the selected horses are summarised in Table 1.

**Table 1:** Clinical signs, serology and PCV of selected horses

Parameter used for selection	Mares		Stallions	
	N02	N59	N01	N6A
Oedema of the vulva	+	+	NA	NA
Depigmentation around genitalia	+	–	+	+
Oedema of mammary gland	+	–	NA	NA
Difficulty in walking with straddle gait	+	+	+	–
Emaciation	+	+	+	+
CATT/*T. evansi*	+	+	+	+
PCV (%)	26	29	24	30

### PCR

**Fig. 2:**
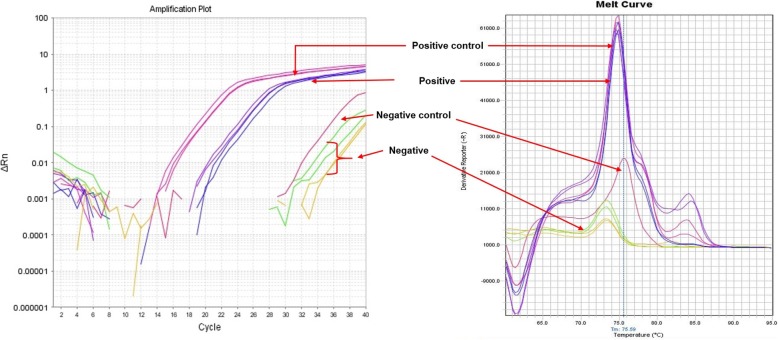
Real-time PCR: Amplification plot and melt curve to show positivity of the tested samples in relation to positive and negative controls

The presence of the parasite in a number of organs was confirmed by real- time PCR on DNA extracted from tissues collected at necropsy (Table 2). Positive results were demonstrated by photo data of amplification plot and melt curve (Fig. 2).

**Table 2:** Results of real-time PCR of necropsy samples

Name of Tissue	Mares		Stallions	
	N02	N59	N01	N6A
Nervous system				
Brain	+	+	+	+
Spinal cord	+	+	+	+
Caudal nerve	+	–	+	–
Sciatic nerve	+	–	+	–
Cerebrospinal fluid	+	+	+	+
Reproductive system				
Penis	NA	NA	+	+
Testicle	NA	NA	+	+
Epididymis	NA	NA	+	+
Prostate gland	NA	NA	+	+
Vesicular gland	NA	NA	+	–
Bulbourethral gland	NA	NA	+	–
Ampulla	NA	NA	+	+
Urethra	+	–	+	+
Urinary bladder	–	+	ND	+
Vagina	–	+	NA	NA
Vestibule	+	+	NA	NA
Cervix	+	+	NA	NA
Uterus	+	+	NA	NA
Mammary gland	+	+	NA	NA
Others				
Kidney	ND	+	+	+
Heart	+	+	+	+
Pancreas	–	–	+	–
Spleen	+	–	–	+
Blood	–	–	–	–

(+) = PCR positive for trypanosomes, (−) = PCR negative, NA not applicable, ND = not done

**Fig. 3:**
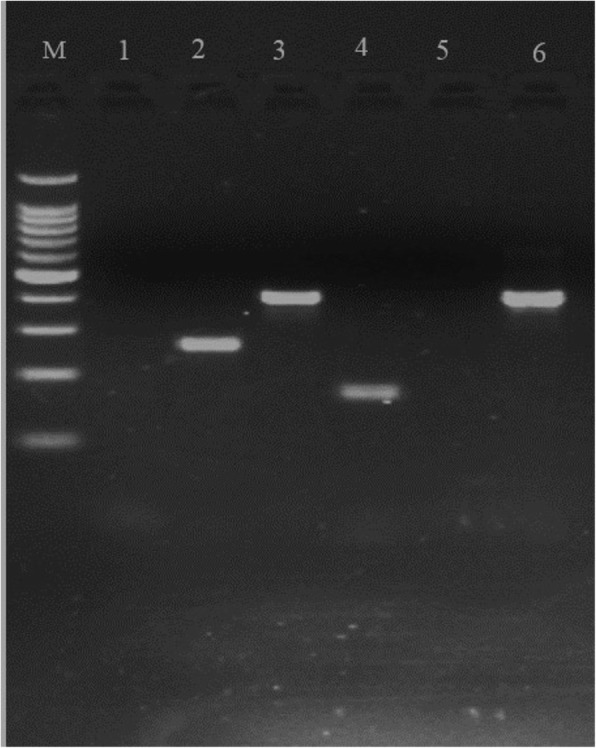
PCR profile of DNA extracted from tissues for the differentiations of *T*. *equiperdum* and *T. evansi* targeting maxicircle and RoTat 1.2 genes. Lane M:100 bp plus marker; lane 1: Blank; lane 2: ND4 (256 bp); lane 3: ND5 (400 bp); lane 4: ND7 (167 bp); lane 5: RoTat1.2 (negative); lane 6: A6 (381bp)

To differentiate between *T. equiperdum* and *T. evansi*, samples from nerve tissues, testicles and vestibules that were positive on real-time PCR using the ITS primer, were further checked by conventional PCR targeting the maxi-circle genes (unique for *T. equiperdum*) and VSG genes (RoTat 1.2) (typical for *T. evansi*) and were all found to be positive for the maxi-circle and negative for the RoTat 1.2 (Fig. 3).

### Post-mortem and histopathology

Necropsy of the naturally dourine diseases horses did not show significant lesions in most of the organs. A small haemorrhage in the spinal cord, some pinpoint white zones in the liver were observed but not consistent in all horses. There was some serous fluid accumulation in the pericardium in one horse. Helminth parasites in the intestine were found in all of the horses.

**Fig. 4:**
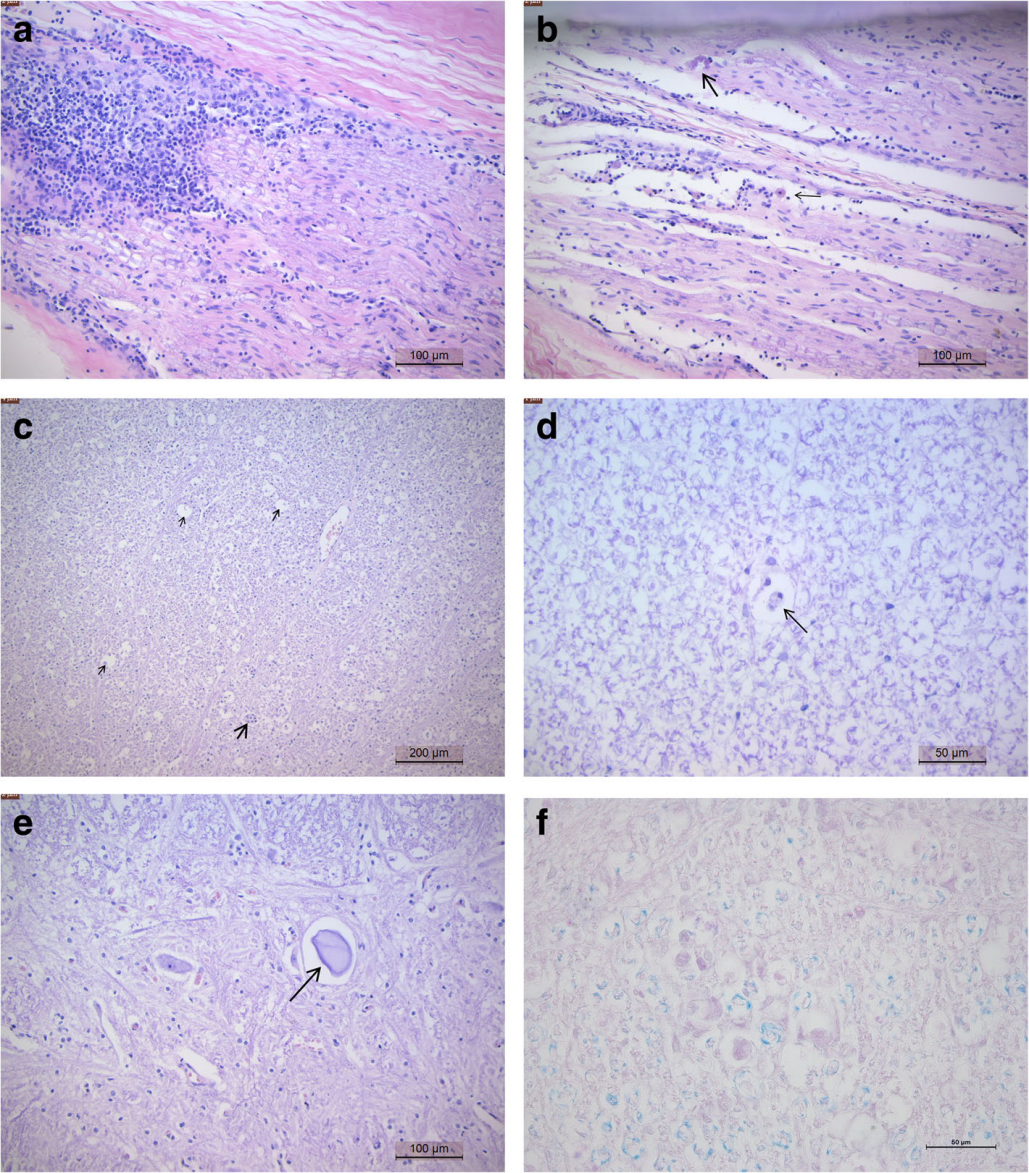
Photomicrograph of the lesions in the nervous system (H/E) (**a**) Peripheral nerve with severe infiltration of lymphocytes and histiocytes (**b**) Peripheral nerve - infiltration of lymphocytes, plasma cells and histiocytes with axonal swelling, fragmentation (large arrow) and phagocytosis of debris (small arrow) (**c**) Spinal cord white matter - empty myelin sheaths (small arrows), with phagocytosis of debris (large arrow) (**d**) Spinal cord white matter- swollen myelin sheath with phagocytosis (arrow) (**e**) Spinal cord grey matter - focal neuronal degeneration (arrow), normal neuronal cell body to the left for comparison (**f**) Photomicrograph showing affected spinal cord white matter with overall reduction in blue staining indicates loss of myelin (demyelination). Note empty spaces with central swollen axons (pink spheroids)

Histopathological lesions were most severe in the peripheral nerves (and associated ganglia) and consistent in all animals. In the affected nerves, multifocal infiltration of lymphocytes, plasma cells and macrophages were found between the axons of nerve fascicles, with variable axonal swelling and fragmentation (Fig. 4a-b).

In the spinal cord, lesions were located in the white matter and consisted of multifocal axonal degeneration manifested by scattered empty myelin sheaths with histiocytes and phagocytosis of debris, seldom with spheroids. Severe axonal degeneration occurred in the dorsal funiculus in the posterior part of the spinal cord (Fig. 4c-d). In the grey matter, histopathological lesions were minimal and confined to scattered neuronal degeneration (Fig. 4e). At the affected spinal cord at the junction of white matter and grey matter there was a reduction in overall blue staining of the white matter indicating demyelination (Fig. 4f). No lesions were seen in the cerebrum, cerebellum or brain stem except for rare mild axonal degeneration.

**Fig. 5:**
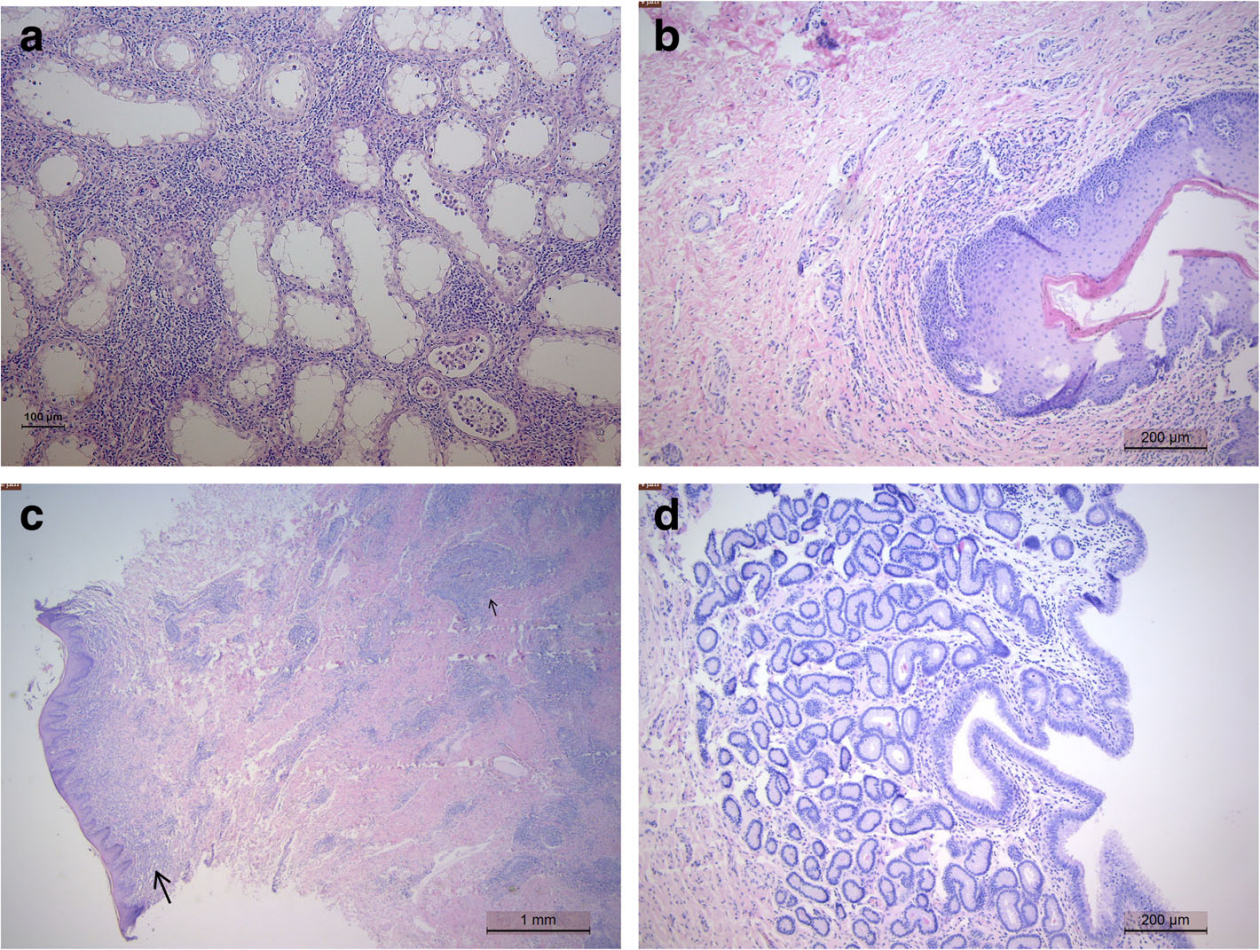
Photomicrograph of the lesions in the genetalia of dourine diseased horses (H/E) (**a**) Testicle - chronic orchitis with absence of spermatogenesis and infiltration of lymphocytes and plasma cells (**b**) Glans penis - infiltration of lymphocytes, plasma cells and histiocytes (**c**) Vestibular mucosa - superficial diffuse (large arrow) and deep nodular (small arrow) infiltration of lymphocytes, plasma cells and histiocytes (**d**) Endometrium - infiltration of lymphocytes

Histopathological examination of the male reproductive system showed severe diffuse orchitis with interstitial infiltration of lymphocytes, plasma cells and histiocytes. Inflammation with interstitial fibroblasts and diffuse degeneration of seminiferous tubules with absence of spermatogenesis were present (Fig. 5a). Also, the interstitium of the epididymis was infiltrated by lymphocytes, plasma cells and histiocytes. In the superficial genital mucosa (glans penis), there was frequent lymphoplasmacytic and histiocytic inflammation (Fig. 5b). No lesions were seen in the accessory sexual glands.

In the female reproductive tract, most lesions were found in the vaginal and vestibular mucosa consisting of nodular infiltrations of lymphocytes, plasma cells and histiocytes associated with erosions (Fig. 5c). In the uterus lymphocytic inflammation was confined to the endometrium (Fig. 5d).

Kidneys showed no lesions except foci of mineralization at the level of the tubules in one horse. In the spleen, there were many macrophages with hemosiderin and the white pulp was reactive. In the liver multifocal necrotic cell debris surrounded by epithelioid macrophages, lymphocytes and eosinophils and a fibrous capsule (granuloma) were observed. In the intestines, infiltration of lymphocytes, eosinophils and macrophages in the lamina propria and submucosa were seen. Macrophages were also found multifocal in the cranial mesenteric artery. The mesenteric lymph nodes were oedematous in subcortical and medullary sinuses with mild infiltration of macrophages.

## Discussion

During selection of the four dourine diseased horses for the necropsy study, 100 dourine suspected horses were examined. The signs observed in this field survey were similar to those reported before [6, 7, 11], including swelling of genitalia and udder, nervous signs, emaciation and depigmentation of the perineal skin. Skin plaques or wheals, which were previously regarded as pathognomonic symptoms of dourine [10, 11] or as rare symptoms, only present in a few cases [6, 7, 18], were not observed in this study. Differences in symptomatology might be due to the difference in the clinical stage, the strain of the parasite or breed of horse [19, 20].

It was not possible to isolate the parasite through the buffy coat examination (Woo test) in any of the 4 horses. *T. equiperdum* is considered a tissue parasite and can rarely be found in the blood [19] resulting in low parasitaemia in chronical cases [21]. Despite these difficulties, parasites were isolated from blood [9, 22] and mammary secretions [17] of clinically sick horses. Isolation from the urethral tract [23] and semen [24] of dourine diseased stallions was also reported.

Demonstration of *T. equiperdum* DNA in tissues by PCR using the ITS primer, with a reported sensitivity for *Trypanozoon* of 10 pg (100–200 copy number of a DNA sequence) [25–27] was successful in the brain, spinal cord, peripheral nerves of the hind quarter, uterus, ovaries, vagina, testicles, kidneys, heart and liver. Pascucci et al. [17] detected already parasite DNA by real-time PCR in vaginal swabs, joint fluid, lymph nodes and mammary secretions using a set of primers (Tb177F and Tb177R) directed for a common sequence in *Trypanozoon*. Demonstration of *T. equiperdum* DNA in tissues by PCR might significantly facilitate the diagnosis of dourine in live animals on biopsy samples from lymph nodes, skin lesions, vulva and uterus, or from epididymal semen.

To differentiate *T. equiperdum* from *T. evansi*, DNA samples from these naturally infected horses, positive on real-time PCR using the ITS primer, were run again over more specific PCRs targeting maxicircle genes (specific to *T. equiperdum*) which include the ATPase subunit 6 (A6) and three NADH-dehydrogenase subunits (ND4, ND5, ND7) and RoTat 1.2 PCRs targeting VSG genes (specific to *T. evansi*). All extracts were positive for all four maxicircle genes but negative for the RoTat 1.2 VSG genes. Ethiopian *T. evansi* stocks and *T. evansi* type A RoTat 1.2 and *T. evansi* type B KETRI 2479 are negative for all maxicircle genes [28], while *T. equiperdum* Dodola 940, isolated from dourine horses in the study area [22], is positive for all maxicircle genes similar to the present findings. Diagnosis was possible using extracts from the predilection sites of the parasite in diseased horses. The lack of species-specificity of the CATT-test could also be demonstrated when Trypanosome free horses were infected experimentally with *T. equiperdum* Dodola 943 in another experiment and appeared to become positive when tested with the CATT/*T. evansi* test. This can be explained by the fact that CATT/*T. evansi* reagent consists of acetone/formaldehyde fixed coomassie-stained pure parasites (*T. evansi*) which may contain cross-reacting epitopes with other Trypanosomes in particular *T. equiperdum*. It is a crude antigene that has *T. evansi* specific (VSG RoTat 1.2) and nonspecific epitopes (shared by the *Trypanozoon*) [29, 30].

To exclude the Ethiopian *T. equiperdum* from tsetse transmitted *T. brucei* based on the presence of this target gene, Dodola (Ethiopia), where the current dourine diseased horses came from, is out of the tsetse belt [31, 32]. In Ethiopia, trypanosomes such as *T. vivax*, *T. congolense*, *T. brucei*, *T. evansi* and *T. equiperdum* are the common causes of African animal trypanosomosis [33]. *T. congolense* and *T. brucei* are only important in the tsetse-infested areas whereas *T. evansi* and *T. equiperdum* are found out of the tsetse belt. *T. vivax* can be found everywhere both in tsetse-infested and tsetse-free areas except in areas with an altitude of > 2500 m above sea level [33–35].

Literature on pathological lesions caused by *T. equiperdum* in horses is scanty. In this study, no lesions in the parenchymatous organs were seen except for small haemorrhages in the spinal cord and lungs and some pinpoint white zones in the liver. Earlier it was reported that the spleen was congested with subcapsular blood suffusion and bloody nodules thickened connective tissue and abundant synovial fluid in the tarsometatarsal joints [16, 17]. The lesions in the non-reproductive organs in this study were inconsistent with previous reports [17] where no lesions were observed in the parenchymatous organs except the spleen. These lesions, however, were not considered as specific lesions of dourine. The histopathological changes, especially cellular infiltration and degenerative changes of the spinal nerves and ganglia involving the obturator and sciatic nerves, were also mentioned in previous reports [15] although not in all [16, 17]. Neuritis associated with vacuolation and demyelination of the facial nerve with infiltrating cells primarily identified as lymphocytes plasma cells and some macrophages were also reported from dourine diseased horses in Mongolia. Few axonal swellings were noted within the demyelinating foci [36].

In contrast to horses infected by *T. evansi* [37, 38] or *T. brucei* [39, 40], histopathological changes in the brain induced by *T. equiperdum* are minimal. However, lesions of the spinal cord, especially to the lumbar and sacrococcygeal part, are more severe than those in the brain. The localisation of these lesions might be associated with the mechanism of cerebrospinal fluid (CSF) drainage. CSF leaves the subarachnoid spaces through the arachnoid villi, reaching the dural sinuses and the bloodstream. Absorption occurs at the sub-arachnoid vessels. Finally, the CSF flows along the spinal nerves into the lymphatic stream [41]. The trypanosomes, their extracellular products and antigenic components in the CSF drain along the spinal nerves and might elicit a host response at these sites.

The microscopic lesions observed in the genital tract of mares, as infiltration of mononuclear cells, periglandular inflammation of vulva and vagina, were not reported earlier. However perivascular inflammation in the uterine submucosa and oedema of the skin overlying the udder are in line with previous observations [17].

Depigmentation around the perineum is described to be characteristic for dourine [7, 11, 19]. In our study, this symptom was observed in one of the mares, microscopically characterized by lymphoplasmocytic inflammation. Pigment loss could be secondary to necrosis of the epidermis containing the melanocytes with fibrocyte infiltration. Gizaw et al. [42] indicated that it was a sequel of dermatitis with hydropic degeneration and necrosis of the keratinocytes in the stratum spinosum and basal cells including the melanocytes within the epidermis. Since melanin is stored in melanocytes damage can cause loss of melanin resulting in depigmentation [43]. Transient clinical EHV3 infections might give similar depigmented spots at the perineum [44] and it was unknown in the current study if these spots were already present before the *T. equiperdum* infection.

Intestinal and mesenteric lymph nodes showing non-specific reactivity might be a response to the intestinal parasites. Multifocal hepatitis in the liver, plasma cell inflammation of the renal pelvis and lymph node reactivity were also reported in horses suffering dourine [17]. The haemosiderin deposition in the spleen might indicate a role of the spleen in the destruction of red blood cells during trypanosomosis [17]. Infiltration of inflammatory cells, especially lymphocytes, plasma cells and some macrophages is a hallmark of chronic inflammation [45] and was seen in most tissues of the nervous and reproductive tract.

## Conclusions

*T. equiperdum* spread to many tissues with histopathological features in the peripheral nerves and the genital organs. Lesions in the reproductive organs, the distal spinal cord and the peripheral nerves with massive infiltration of mononuclear cells revealed an immunological response of the host to the parasite (or products) and explain the clinical diagnostic observations of incoordination in the hind legs, whilst its presence in the genital tract exemplifies the venereal transmission.

## Methods

### Study area

The study was carried out in the Arsi-Bale highlands (Ethiopia), a dourine endemic area (6.58°N latitude and 39.18°E longitude at 2400 m above mean sea level). Agriculture is the mainstay of the livelihood of people and the leading economic activity of the area.

### Study animals

Animals considered were adult horses suspected of *T. equiperdum* infection by either exhibiting clinical signs or with a history of dourine, serologically positive by Card Agglutination test for trypanosomes (CATT/*T. evansi*) and with low PCV values. They were kept under a traditional extensive management system of free grazing in the communal lands. Two mares and two stallions were purchased from the local farmers and euthanized and necropsy was performed according to standard procedures [46] including histopathologic [47] and PCR [26, 27] examinations of a variety of tissue samples using standard methods. To clarify the specific tissue structures of axons of the spinal cord whether they were demyelinated or not, luxol fast blue staining was performed [48]. Horses were euthanized humanely by intravenous administration of over dose sodium pentobarbital (50 mg/kg) after sedated with xylazine at a dose of 1 mg/kg body weight. The procedures were approved by the Ethical Review Committee of Addis Ababa University, College of Veterinary Medicine and Agriculture (Permit No: VM/ERC/004/07/015).

### Blood collection

Blood samples were collected using vacutainer tubes (Golden Vac™, Zhenjiang Gonggdong medical technology Co. Ltd.), for serological-tests and parasitological examination.

### Parasitological examination

Haematocrit centrifugation was used to isolate the parasite from blood. Capillary tubes, with 50 μl blood were centrifuged for 5 min at 3000 g. The buffy coat plasma interface layer was examined (× 100) to look for parasites [49, 50]. The PCV was measured using a microhaematocrit reader (Hawksley, UK).

### Serological examination

The CATT*/T. evansi* (Institute of Tropical Medicine, Antwerp, Belgium) serological test was performed as described previously [7, 9, 51].

### DNA extraction and PCR

DNA extraction was performed using a DNeasy Blood and Tissue DNA Extraction Kit (Qiagen, Germany) [52]. A tissue of 25 mg (10 mg for spleen) was cut in to pieces and added in to a 1.5 ml microcentrifuge tube containing 180 μl buffer ATL and 20 μl proteinase K. The tissue sample was completely lysed at a temperature of 56 °C for up to 3 h. DNA was extracted into 200 μL elution buffer AE according to the manufacturer’s instructions. One sample per organ was used to extract the DNA unless the purity based on 260/280 and 260/230 ratios in a NanoDrop Spectrophotometry was disrupted. After extraction, DNA was stored at − 20 °C until analysis. The DNA concentrations were measured using the Nanodrop ND-2000 UV- Vis spectrophotometer (Nanodrop Technologies, USA). The quality was further checked by PCR using Cytochrome b primer targeting the host DNA.

The DNA samples were tested by real-time PCR targeting the internal transcribed spacer regions (ITS1) of trypanosomes with forward primer 5’TGTAGGTGAACCTGCAGCTGGATC3’ and reverse primer 5’CCAAGTCATCCATCGCGACACGTT3’ [26] resulting in fragments of approximately 450 bp [26, 27]. The method was done on a Step One Plus Real-Time PCR System (Life Technologies) following detailed procedures of our previous work [53]. Samples were considered positive when the observed amplification and melting curve are similar to positive control and negative samples are also included (Fig. 2).

To differentiate *T. equiperdum* from *T. evansi* more PCR were performed targeting the maxicircle of *T. equiperdum* which are not found in *T. evansi* [54]. A set of PCRs targeting VSG genes (RoTat 1.2), maxicircle genes (ND4, ND5, ND7 and A6), with different sets of primers and reaction mixtures (Table 3) were used to differentiate *T. equiperdum* from *T. evansi* [28]. Amplifications in a conventional PCR were carried out in 200 μl thin-wall PCR tubes in a Veriti thermal cycler 96 (Applied Biosystems) with two kinds of reaction mixtures (Table 3). Where applicable, the published PCR protocols were adjusted to the requirements of the GoTaqG2 Flexi DNA polymerase (Promega Corporation USA). Ten microliters of the amplified product was used for electrophoresis in 2% agarose gel at 85 V for 35 min and stained with ethidium bromide for UV visualization.

**Table 3:** PCR conditions used to differentiate *T. equiperdum* and *T. evansi*

Target	Primers	Primer sequences	Amplicon length (bp)	Reaction mixture^a^	Cycling conditions	Adapted from
VSG RoTat 1.2	ILO7957 ILO8091	5′-GCC ACC ACG GCG AAA GAC-3′ 5′-TAA TCA GTG TGG TGT GC-3′	488	a	95 °C for 5 min and 35 cycles of 30 s at 94 °C, 30 s at 58 °C, 30 s at 72 °C and final extension for 5 min at 72 °C	[55]
VSG RoTat 1.2	RoTat1.2-F RoTat1.2-R	5′-GCGGGGTGTTTAAAGCAATA-3′ 5′-ATTAGTGCTGCGTGTGTTCG-3’	205	a	95 °C for 15 min and 40 cycles of 30 s at 94 °C, 30 s at 59 °C, 30 s at 72 °C and final extension for 5 min at 72 °C.	[56]
Maxicircle A6	Forward Reverse	5′AAAAATAAGTATTTTGATATTATTAAAG-3′ 5′-TATTATTAACTTATTTGATC-3′	381	b	95 °C for 5 min and 30 cycles of 94 °C for 1 min, 54 °C for 1 min and 72 °C for 30s followed by a final elongation step at 72 °C for 8 min	[57]
Maxicircle ND4	Forward Reverse	5′-TGTGTGACTACCAGAGAT-3′ 5′ -ATCCTATACCCGTGTGTA-3	256	b	Idem as above	[57]
Maxicircle ND5	Forward Reverse	5′-TGGGTTTATATCAGGTTCATTTATG-3′ 5′ -CCCTAATAATCTCATCCGCAGTACG-3′	400	b	Idem as above	[58]
Maxicircle ND7	Forward Reverse	5′-ATGACTACATGATAAGTA-3′ 5′ -CGGAAGACATTGTTCTACAC-3′	167	b	Idem as above	[57]

^a^Reaction mixture (a): 25 μl containing 25 ng DNA, 1x Green GoTaq G2 Flexi buffer, 2 mM of MgCl2, 0.2 mM of each dNTPs, 0.5 μM of each primer, 1.25 U GoTaqG2 Flexi DNA polymerase. Reaction mixture (b): 25 μl containing 25 ng DNA, 1x Green GoTaq G2 Flexi buffer, 2 mM of MgCl2, 0.2 mM of each dNTPs, 1 μM of each primer, 1.25 U GoTaqG2 Flexi DNA polymeras

## Abbreviations

CATT: Card Agglutination Test for Trypanosomes
CSA: Central Statistics Authority
PCR: Polymerase Chain Reaction
PCV: Packed Cell Volume
VSG RoTat: Variable Surface Glycoprotein Rhodense Type

## References

[1] CSA (2017). Agricultural sample survey report on livestock and livestock characteristics, volume II, Statistical bulletin 585 Addis Ababa, Ethiopia.

[2] Swann WJ (2006). Improving the welfare of working equine animals in developing countries. Appl Anim Behav Sci.

[3] Guyo S, Legesse S, Tonamo A (2015). A review on welfare and management practices of working equines. Glob J Anim Sc Livers Prod Anim Breed.

[4] Van Dijk L, Duguma BE, Hernández Gil M, Marcoppido G, Ochieng F, Schlechter P, Starkey P, Wanga C, Zanella A (2014). Role, impact and welfare of working (traction and transport) animals. FAO Animal Production and Health Report (FAO) eng no 5.

[5] Stringer A, Lunn DP, Reid S (2015). Science in brief: report on the first Havemeyer workshop on infectious diseases in working equids, Addis Ababa, Ethiopia. Equine Vet J.

[6] Alemu T, Luckins AG, Phipps LP, Reid SWJ, Holmes PH (1997). The use of enzyme linked immunosorbent assays to investigate the prevalence of *Trypanosoma equiperdum* in Ethiopian horses. Vet Parasitol.

[7] Hagos A, Abebe G, Büscher P, Goddeeris BM, Claes F (2010). Serological and parasitological survey of dourine in the Arsi–bale highlands of Ethiopia. Trop Anim Health Prod.

[8] Hagos A, Degefa G, Yacob H, Fikru R, Alemu T, Feseha G, Claes F, Goddeeris BM (2010). Seroepidemiological survey of Trypanozoon infection in horses in the suspected dourine-infected bale highlands of the Oromia region, Ethiopia. Rev Sci Tech Off Int Epiz.

[9] Gari FR, Ashenafi H, Tola A, Goddeeris BM, Claes F (2010). Comparative diagnosis of parasitological, serological and molecular tests in dourine-suspected horses. Trop Anim Health Prod.

[10] OIE (2013). Dourine, Chapter 2.5.3. OIE Terrestrial Manual Version adopted by the World Assembly of Delegates of the OIE in May 2013.

[11] Vulpiani MP, Carvelli A, Giansante D, Iannino F, Paganico D, Ferri N (2013). Reemergence of dourine in Italy: clinical cases in some positive horses. J Equine Vet Sci..

[12] Clausen PH, Gebreselassie G, Abditcho S, Mehlitz D, Staak C (1998). Detection of trypanosome DNA in serologically positive but aparasitaemic horses suspected of dourine in Ethiopia. Tokai J Exp Clin Med.

[13] Clausen PH, Chuluun S, Sodnomdarjaa R, Greiner M, Noeckler K, Staak C, Zessin KH, Schein E (2003). A field study to estimate the prevalence of *Trypanosoma equiperdum* in Mongolian horses. Vet Parasitol.

[14] Ahmed Y, Hagos A, Merga B, Van Soom A, Duchateau L, Goddeeris BM, Govaer J (2018). *Trypanosoma equiperdum* in the horse-a neglected threat? Vlaams Diergeneeskd Tijdschr.

[15] Barrowman PR (1976). Observations on the transmission, immunology, clinical signs and chemotherapy of dourine (*Trypanosoma equiperdum* infection) in horses, with special reference to cerebro-spinal fluid. Onderstepoort J Vet Res.

[16] Scacchia M, Cammà C, Di Francesco G, Di Provvido A, Giunta R, Luciani M, Marino AMF, Pascucci I, Caporale V (2011). A clinical case of dourine in an outbreak in Italy. Vet Ital.

[17] Pascucci I, Di Provvido A, Cammà C, Di Francesco G, Calistri P, Tittarelli M, Ferri N, Scacchia M, Caporale V (2013). Diagnosis of dourine in outbreaks in Italy. Diagnosis of dourine in outbreaks in Italy. Vet Parasitol.

[18] Claes F, Agbo EC, Radwanska M, Te Pas MFW, Baltz T, De Waal DT, Goddeeris BM, Claassen E, Büscher P (2003). How does Trypanosoma equiperdum fit into the *Trypanozoon* group? A cluster analysis by RAPD and multiplex-endonuclease genotyping approach. Parasitology.

[19] Stephen LE (1986). Trypanosomiasis: a veterinary perspective.

[20] Ricketts S, McGladdery A, Crowhurst J, Newton R (2011). Dourine, an emerging venereal threat to European horses. Equine Quarterly Disease Surveillance report.

[21] Zablotskij VT, Georgiu C, De Waal T, Clausen PH, Claes F, Touratier L (2003). The current challenges of dourine: difficulties in differentiating *Trypanosoma equiperdum* within the subgenus *Trypanozoon*. Rev sci Tech Off int Epiz.

[22] Hagos A, Goddeeris BM, Yilkal K, Alemu T, Fikru R, Yacob HT, Feseha G, Claes F (2010). Efficacy of Cymelarsan® and Diminasan® against *Trypanosoma equiperdum* infections in mice and horses. Vet Parasitol.

[23] Suganuma K, Narantsatsral S, Battur B, Yamasaki S, Otgonsuren D, Musinguzi PS, Davaasuren B, Battsetseg B, Inoue N (2016). Isolation, cultivation and molecular characterization of a new *Trypanosoma equiperdum* strain in Mongolia. Parasit Vector.

[24] Ahmed Y, Hagos A, Merga B, Alemu T, Van Soom A, Duchateau L, Goddeeris B, Govaere J (2017). Infectiousness of equine semen in the prepatent phase of dourine. Reprod Dom Anim.

[25] Desquesnes M, Bosseno MF, Brenière SF (2007). Detection of Chagas infections using *Trypanosoma evansi* crude antigen demonstrates high cross-reactions with *Trypanosoma cruzi*. Infect Genet Evol.

[26] Fikru R, Goddeeris BM, Delespaux V, Moti Y, Tadesse A, Bekana M, Claes F, De Deken R, Büscher P (2012). Widespread occurrence of *Trypanosoma vivax* in bovines of tsetse-as well as non-tsetse-infested regions of Ethiopia: a reason for concern. Vet Parasitol.

[27] Njiru ZK, Constantine CC, Guya S, Crowther J, Kiragu JM, Thompson RCA, Dávila AMR (2005). The use of ITS1 rDNA PCR in detecting pathogenic African trypanosomes. Parasitol Res.

[28] Birhanu H, Gebrehiwot T, Goddeeris BM, Büscher P, Van Reet N (2016). New Trypanosoma evansi type B isolates from Ethiopian dromedary camels. PLoS Negl Trop Dis.

[29] Claes F, Verloo D, De Waal DT, Urakawa T, Majiwa P, Goddeeris BM, Büscher P (2002). Expression of RoTat 1.2 cross-reactive variable antigen type in *Trypanosoma evansi* and *T. equiperdum*. Ann N Y Acad Sci.

[30] Birhanu H, Fikru R, Said M, Kidane W, Gebrehiwot T, Hagos A, Alemu T, Dawit T, Berkvens D, Goddeeris BM, Büscher P. Epidemiology of Trypanosoma evansi and Trypanosoma vivax in domestic animals from selected districts of Tigray and Afar regions, Northern Ethiopia. Parasite Vector. 2015;8:212. 10.1186/s13071-015-0818-1 PMC4403896 25889702

[31] Abebe G (2005). Trypanosomosis in Ethiopia. *Ethiop*. J Biol Sci.

[32] Dagnachew S, Bezie M, Terefe G, Abebe G, Barry JD, Goddeeris BM (2015). Comparative clinico-haematological analysis in young zebu cattle experimentally infected with *Trypanosoma vivax* isolates from tsetse infested and non-tsetse infested areas of Northwest Ethiopia. Acta Vet Scand.

[33] Dagnatchew Z. Trypanosomiasis in Ethiopia. In: International Symposia on Veterinary Epidemiology and Economics proceedings, ISVEE 3: Veterinary epidemiology and economics, proceedings of the 3rd international symposium. Arlington, Virginia, USA; 1982. p. 467–73. http://www.sciquest.org.nz/node/61198.

[34] Abebe G, Yilma J (1996). Trypanosomosis: a threat to cattle production in Ethiopia. Rev Med Vet.

[35] Hadush AB (2016). Trypanosoma evansi in northern Ethiopia: epidemiology, diversity and alternative diagnostics (doctoral dissertation, KU Leuven).

[36] Mungun-Ochir B, Horiuchi N, Altanchimeg A, Koyama K, Suganuma K, Nyamdolgor U, Watanabe K, Baatarjargal P, Mizushima D, Battur B, Yokoyama N, Battsetseg B, Inoue N, Kobayashi Y. Polyradiculoneuropathy in dourine-affected horses. Neuromuscul Disord. 2019. 10.1016/j.nmd.2019.03.005 In press. 10.1016/j.nmd.2019.03.005 31101461

[37] Seiler RJ, Omar S, Jackson ARB (1981). Meningoencephalitis in naturally occurring *Trypanosoma evansi* infection (Surra) of horses. Vet Pathol.

[38] Rodrigues A, Fighera RA, Souza TM, Schild AL, Barros CSL (2009). Neuropathology of naturally occurring *Trypanosoma evansi* infection of horses. Vet Pathol.

[39] Losos GJ, Ikede BO (1972). Review of pathology of diseases in domestic and laboratory animals caused by *Trypanosoma congolense*, *T. vivax, T. brucei, T. rhodesiense and T. gambiense*. Vet Pathol.

[40] Kingston D, Rodgers J, Sharpe S, Berman K, Morrison L, Kenned P, Bradley B, Sutton DGM (2016). Equine central nervous system trypanosomosis in the Gambia is caused by genetically diverse populations of *Trypanosoma brucei* parasites. J Equine Vet Sci.

[41] Natalini CC (2010). Spinal anesthetics and analgesics in the horse. Veterinary clinics: equine practice.

[42] Gizaw Y, Megersa M, Fayera T (2017). Dourine: a neglected disease of equids. Trop Anim Health Prod.

[43] Myers RK, Mc Gavin MD, McGavin MD, Zachary JF (2006). Cellular and Tissue Responses to Injury. Pathologic Basis of Veterinary Disease.

[44] Blanchard T, Kenney R, Timoney P (1992). Venereal diseases. Vet Clin North Am Equine Pract.

[45] Jones TC, Hunt RD, King NW. Veterinary pathology. 6th ed. Baltimore: Williams and Wilkins; 1997. p. 177–96.

[46] Whitwell K (2009). Post-mortem examination of horses. In Practice.

[47] Slaoui M, Fiette L. Histopathology procedures: from tissue sampling to histopathological evaluation. Methods Mol Biol. 2011;691:69–82. 10.1007/978-1-60761-849-2_4 20972747

[48] Klüver H, Barrera E (1953). A method for the combined staining of cells and fibers in the nervous system. J Neuropathol Exp Neurol.

[49] Woo PTK (1970). The haematocrit centrifuge technique for the diagnosis of African Trypanosomosis. Acta Trop.

[50] Reid SA, Husein A, Copeman DB (2001). Evaluation and improvement of parasitological tests for *Trypanosoma evansi* infection. Vet Parasitol.

[51] Claes F, Verloo D, De Waal DT, Majiwa PA, Baltz T, Goddeeris BM, Büscher P (2003). The expression of RoTat 1.2 variable surface glycoprotein (VSG) in *Trypanosoma evansi* and *T. equiperdum*. Vet Parasitol.

[52] Qiagen. DNeasy Blood and Tissue Handbook 07/2006. QIAGEN GmbH, QIAGEN Strasse 1, vol. 40724. Hilden; 2006. http://diagnostics1.com/MANUAL/General_Qiagen.pdf.

[53] Yasine A, Daba M, Ashenafi H, Geldhof P, Van Brantegem L, Vercauteren G, Demissie T, Bekana M, Tola A, Van Soom A, Duchateau L, Goddeeris B, Govaere J (2019). Tissue (re) distribution of Trypanosoma equiperdum in venereal infected and blood transfused horses. Vet Parasitol.

[54] Brun R, Hecker H, Lun ZR (1998). Trypanosoma evansi and T. equiperdum: distribution, biology, treatment and phylogenetic relationship (a review). Vet Parasitol.

[55] Urakawa T, Verloo D, Moens L, Büscher P (2001). Majiwa PA*Trypanosoma evansi*: cloning and expression in *Spodoptera fugiperda* insect cells of the diagnostic antigen RoTat 1.2. Exp Parasitol.

[56] Claes F, Radwanska M, Urakawa T, Majiw PA, Goddeeris B, Büscher P (2004). Variable surface glycoprotein RoTat 1.2 PCR as a specific diagnostic tool for the detection of *Trypanosoma evansi* infections. Kinetoplastid Biol Dis.

[57] Domingo GJ, Palazzo SS, Wang B, Pannicucci B, Salavati R, Stuart KD (2003). Dyskinetoplastic *Trypanosoma brucei* contains functional editing complexes. Eukaryot Cell.

[58] Dean S, Gould MK, Dewar CE, Schnaufer AC (2013). Single point mutations in ATP synthase compensate for mitochondrial genome loss in trypanosomes. Proc Natl Acad Sci.

